# Investigation of distributive, morphological and audiological factors influencing the long-term aggravation of tinnitus following cochlear implant provision

**DOI:** 10.3389/fneur.2026.1781522

**Published:** 2026-02-25

**Authors:** Friederike Everad, Antje Aschendorff, Ann-Kathrin Rauch, Leonie Fries, Susan Arndt, Manuel Christoph Ketterer

**Affiliations:** Department of Otorhinolaryngology, Medical Center – University of Freiburg, Faculty of Medicine, University of Freiburg, Freiburg, Germany

**Keywords:** cochlear implantation, tinnitus exacerbation, tinnitus burden, tinnitus intensity, tinnitus reduction

## Abstract

**Objectives:**

This prospective study aims to identify factors that may influence the long-term aggravation of tinnitus following cochlear implant (CI) surgery. The variables examined include age, gender, pre- and post-operative residual hearing, duration of deafness, etiology of hearing impairment, cochlear anatomy, as well as the insertion depth and angle of the CI electrode array.

**Methods:**

A total of 65 patients were assessed pre-operatively and at 2 days, 4 weeks, 12 months, and 24 months after CI surgery. Age, gender, duration of deafness, and etiology were recorded through anamnesis. Residual hearing before and after implantation was evaluated using the air conduction (AC) thresholds in four frequencies (500, 1,000, 2,000, and 4,000 Hz) in pure-tone audiometry (PTA-4). Cochlear dimensions (distances and height), insertion depth, and insertion angle were measured using post-operative digital volume tomography. Tinnitus burden and intensity were assessed with the Visual Analog Scale (VAS) and the Tinnitus Questionnaire.

**Results:**

Overall, tinnitus burden significantly decreased during the course of CI treatment. However, 38% of patients reported increased tinnitus intensity on the second post-operative day, with this proportion remaining relatively stable (36%) after 2 years. Meanwhile, 33% of patients experienced an increase of tinnitus burden shortly after implantation, decreasing to 21% 1 year post-operatively. None of the examined factors—including age, gender, residual hearing, duration of deafness, etiology, cochlear anatomy, insertion depth, or insertion angle—were associated with an increased risk of pre-operative tinnitus burden or post-operative tinnitus exacerbation.

**Conclusion:**

Distributive and morphological factors did not significantly influence tinnitus exacerbation before or after CI. Nevertheless, tinnitus symptoms improved significantly over time. Future studies should investigate additional potential contributing factors, such as psychological comorbidities, in the development or persistence of tinnitus following CI.

## Introduction

In the treatment of profound hearing loss and deafness, the provision of a cochlear implant (CI) is the standard of care in both bilateral and unilateral profounded hearing loss and deafness (1, 2). Alongside the well-documented improvement in speech perception in both bilateral and unilateral CI patients, additional factors such as tinnitus (2, 3), preservation of residual hearing (4), and prevention of vertigo (5) have gained increasing interest. Our research group recently presented prospective findings from our study cohort, evaluating the relationship between scalar position, intracochlear trauma due to electrode array dislocation, and post-operative tinnitus development (6). Multiple studies have demonstrated the significant beneficial effect of electrical stimulation of the auditory pathway via CI in tinnitus patients, reducing their tinnitus burden (2, 7–15). Tinnitus has been described as being linked to cochlear deafferentation (2, 16, 17) and neuroplastic changes in the central auditory system. Nevertheless, tinnitus is a highly variable symptom, differing in severity and difficult to measure in hearing-impaired patients. Consequently, it remains unclear why the majority of patients experience tinnitus reduction with CI, while some still report persistent or worsening tinnitus after implantation (2, 18, 19). This prospective study aims to identify factors that may influence the long-term persistance or even aggravation of tinnitus following CI surgery and thus complements the analyses previously conducted by Everad et al. (6). The variables examined include age, gender, pre- and post-operative residual hearing, duration of deafness, etiology of hearing impairment, cochlear height, and electrode array insertion depth and insertion angle.

## Methods

**Figure 1:**
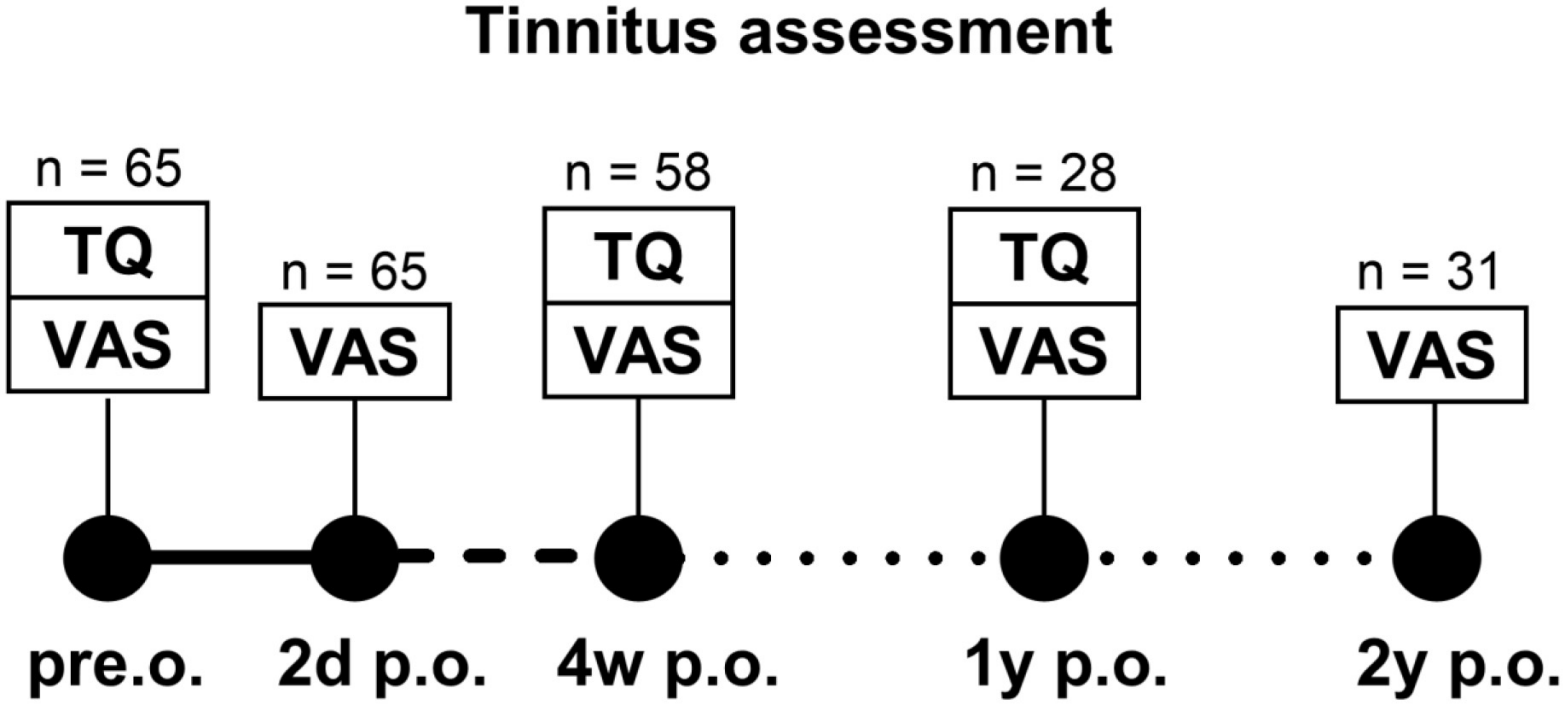
Study flow diagram with number of completed data sets at each time point.

A total of 65 patients were evaluated pre-operatively and at post-operative intervals of 2 days, 4 weeks, 12 months, and 24 months, as detailed in our recently published study (6) (see study flow diagram Figure 1). All consecutive adult patients undergoing cochlear implantation at our center during the recruitment period were screened for eligibility. Tinnitus intensity was assessed using a visual analog scale (VAS), while tinnitus-related distress was measured with the validated German version of the Tinnitus Questionnaire, originally developed by Göbel and Hiller (20). Tinnitus exacerbation was defined as an increase in tinnitus intensity as measured by the VAS and/or an increase in tinnitus-related burden as assessed by the Tinnitus Questionnaire compared to pre-operative baseline. A predefined minimal clinically important difference for these outcome measures in cochlear implant recipients was not available and was therefore not applied. Patient demographics, including age, gender, duration of deafness, and etiology of hearing loss, were obtained through structured anamnesis. Due to the limited number of patients with new-onset post-operative tinnitus, these patients were pooled with those presenting with pre-operative tinnitus for statistical analyses. Pre- and post-operative residual hearing was evaluated using the threshold of the air conducted (AC) pure-tone audiometry in four frequencies (500, 1,000, 2,000, and 4,000 Hz; PTA-4). Cochlear dimensions were measured based on the established distance parameters A and B, as previously described by Escudé et al. (21) and further refined by Ketterer et al. (10, 11, 22, 23). Cochlear height was determined following the method outlined by Ketterer et al. (10, 11). The insertion depth and angle of the cochlear implant electrode array were assessed using digital volume tomography (NewTom 5G / GXL, Hillus Medical Engineering KG, Krefeld, Germany).

Patients were excluded from the study if they had undergone reimplantation, because fibrosis and secondary cochlear trauma may influence outcomes, as demonstrated in previous studies (5, 24). Furthermore, we excluded patients who were younger than 18 years, had cognitive impairment, presented with vestibular schwannoma, or had a history of stapedotomy, labyrinthitis, sacculotomy, or mastoidectomy. In addition, patients with relevant pre-operative residual hearing (defined as PTA4 < 120 dB SPL AC) were excluded from subgroup analysis of the effect of deafness duration on tinnitus exacerbation, as residual acoustic hearing may substantially influence auditory deprivation metrics.

All statistical analyses were performed using the R statistical computing environment (GNU R, Version 3.6.2, R Core Team, Vienna, Austria; http://www.R-project.org). The study protocol received approval from the institutional ethics committee (Ethics Committee Approval No. 129/19) and was prospectively registered in the German Clinical Trials Register (DRKS00034647; http://www.drks.de/). Due to the exploratory and hypothesis-generating nature of the study, no formal *a priori* power calculation was performed. Missing data were handled using complete-case analysis.

## Results

### Distributive factors

#### Evaluation of tinnitus development following cochlear implantation

This study assessed tinnitus progression in 65 adult patients undergoing cochlear implantation, of whom 35 (58%) reported tinnitus pre-operatively by the VAS score. Follow-up attrition increased at later time points. At 2 days post-surgery, 47 patients completed an initial evaluation of tinnitus intensity by the VAS scale: 18 (38%) reported increased tinnitus intensity, 17 (36%) reported no change, and 12 (26%) experienced a reduction (Figure 1). Follow-up assessments were completed by 47 patients at 4 weeks, 28 at 1 year, and 31 at 2 years post-implantation. Differentiation between VAS tinnitus levels with the speech processor on or off was assessed at the 2-year follow-up and showed significant less tinnitus with speech processor on (6).

**Figure 2:**
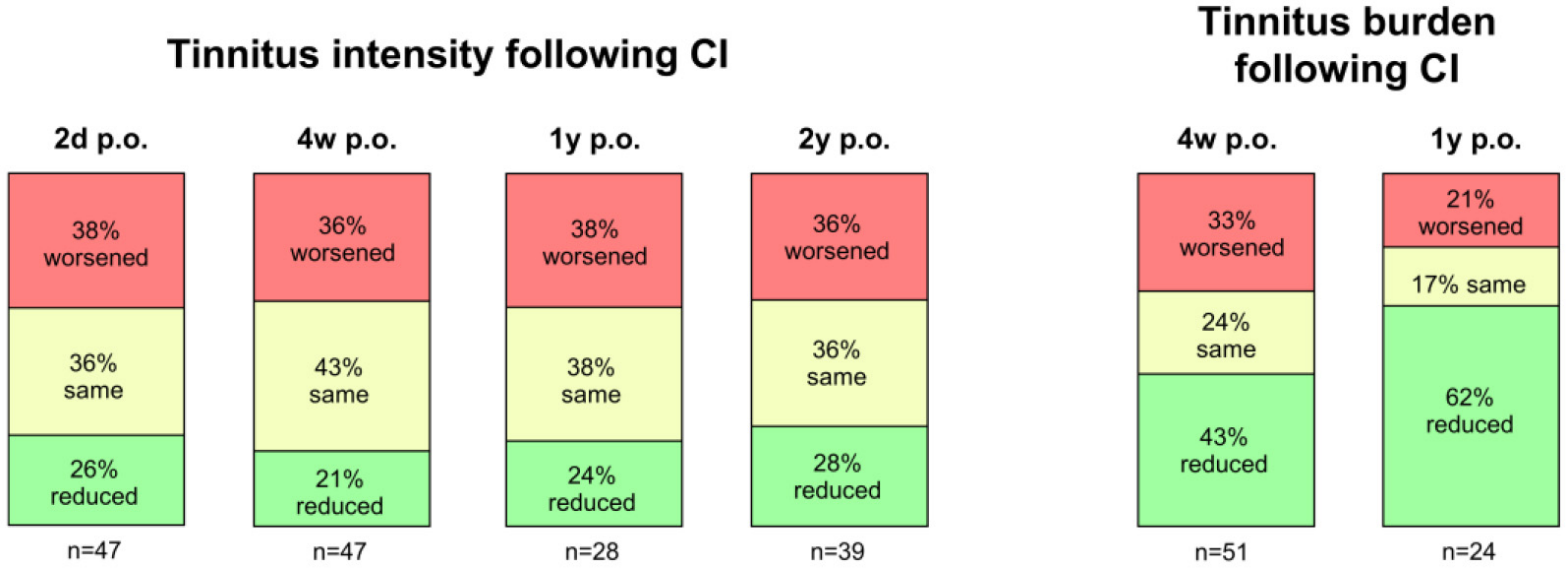
Progression of tinnitus intensity (**left**) and tinnitus burden (**right**) following cochlear implantation over time.

Over time, changes in tinnitus intensity remained relatively stable (Figure 2). However, the subjective burden of tinnitus showed a significant reduction compared to pre-operative levels. At the 1-year follow-up, 24 patients completed the tinnitus questionnaire: 15 (62%) reported a reduced burden, 4 (17%) noted no change, and 5 (21%) experienced an increased burden (Figure 2). The observed discordance between tinnitus intensity and tinnitus-related burden highlights the multifactorial nature of tinnitus perception. While tinnitus loudness may remain unchanged, reductions in tinnitus-related distress may reflect improved coping strategies, habituation, or central adaptation following auditory rehabilitation. This supports the concept that tinnitus intensity and tinnitus burden represent distinct but interrelated dimensions.

#### Potential influence of age on tinnitus exacerbation

**Figure 3:**
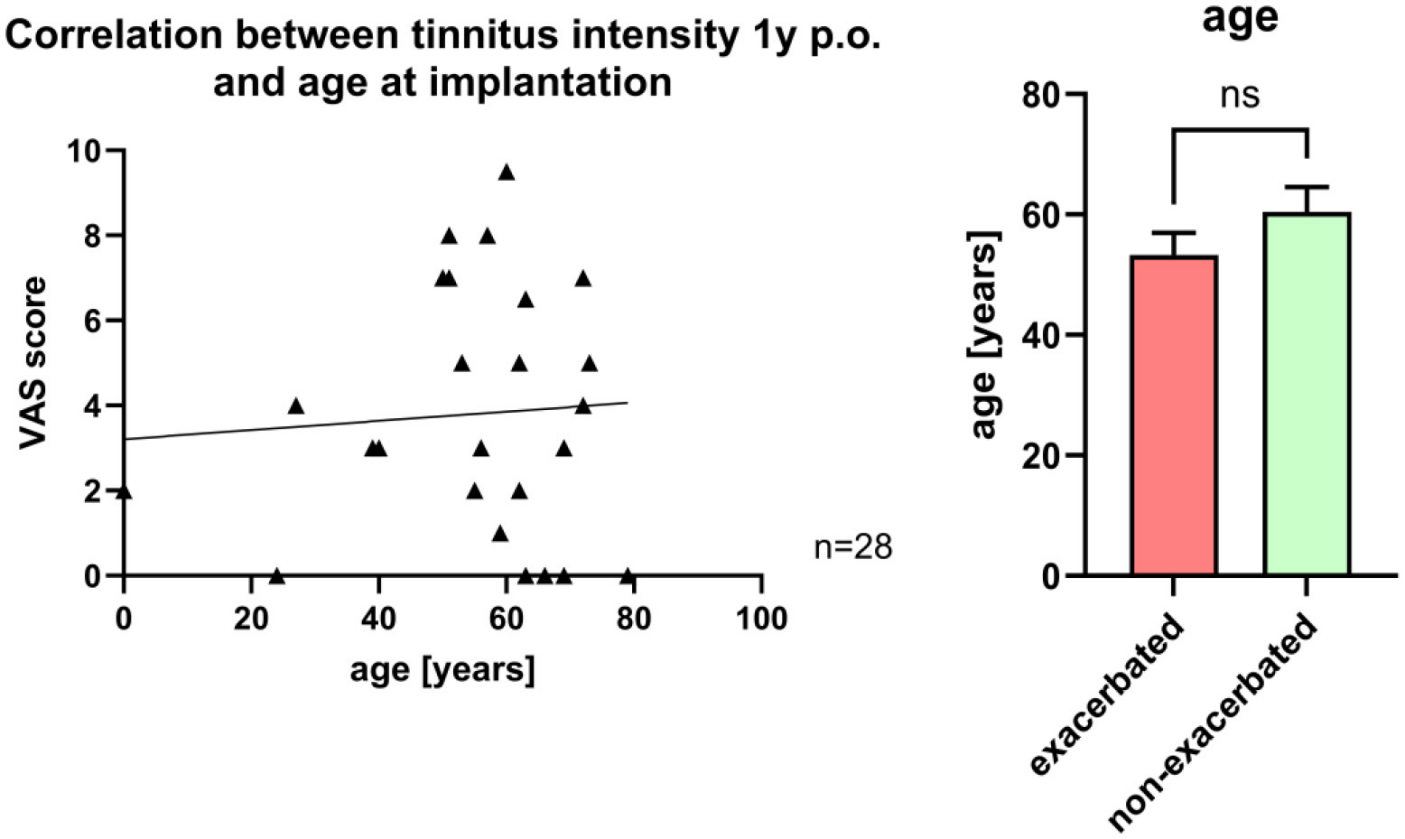
**Left**: correlation between tinnitus intensity and age at implantation one year post-surgery. **Right**: Comparison of ages of patients with exacerbated tinnitus and non-exacerbated tinnitus 1 year post-surgery.

The age of the cohort ranged from 22 to 85 years (mean ± SD: 57.9 ± 14.4 years). No statistically significant differences were observed between the mean age of patients with tinnitus exacerbation and those without, either at 2 days post-surgery (exacerbation: 53.06 ± 17.01 years; non-exacerbation: 59.79 ± 12.98 years) or at one year post-operatively (exacerbation: 53.27 ± 12.22 years; non-exacerbation: 60.46 ± 14.82 years; Figure 3). Additionally, no correlation was found between age and tinnitus intensity at the 1-year follow-up (Figure 3).

#### Potential influence of gender on tinnitus exacerbation

**Figure 4:**
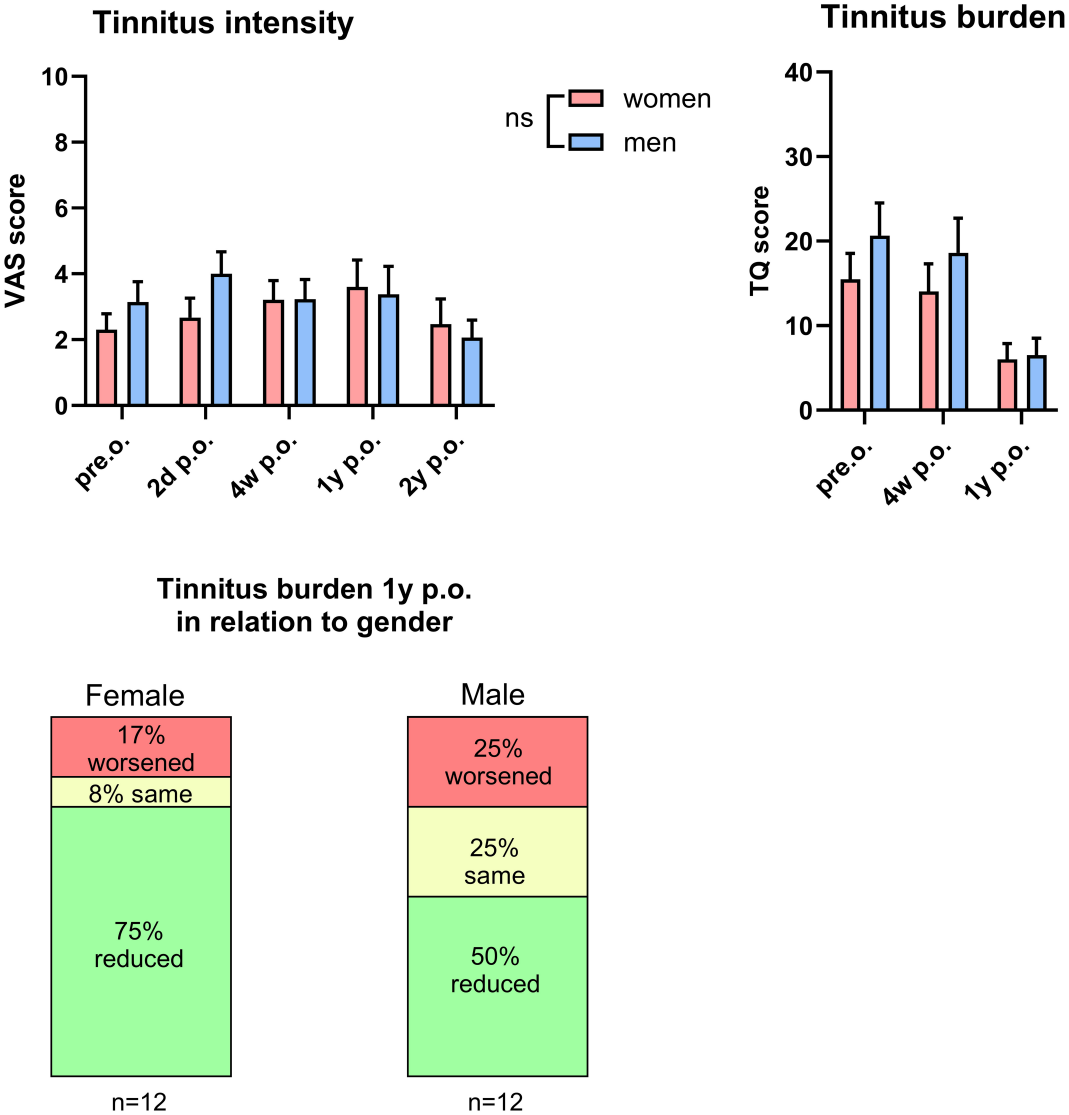
**(a)** Gender comparison of tinnitus intensity (left) and tinnitus burden (right) following cochlear implantation over time. **(b)** Progression of tinnitus burden 1 year post-surgery in female and male patients.

The study cohort comprised 33 female and 27 male patients. Pre-operatively, no significant differences were observed between sexes in tinnitus intensity or burden (VAS: females 2.3 ± 0.47 vs. males 3.6 ± 0.61; TQ: females 15.5 ± 3.1 vs. males 20.7 ± 3.8; Figure 4a). Follow-up evaluations likewise revealed no statistically significant sex differences in VAS or TQ scores (Figure 4a). Interestingly, at 1 year post-surgery, a reduction in tinnitus burden was observed in 75% of females and 50% of males (Figure 4b), suggesting a non-significant trend toward greater improvement in females.

#### Potential influence of duration of deafness on tinnitus exacerbation

**Figure 5:**
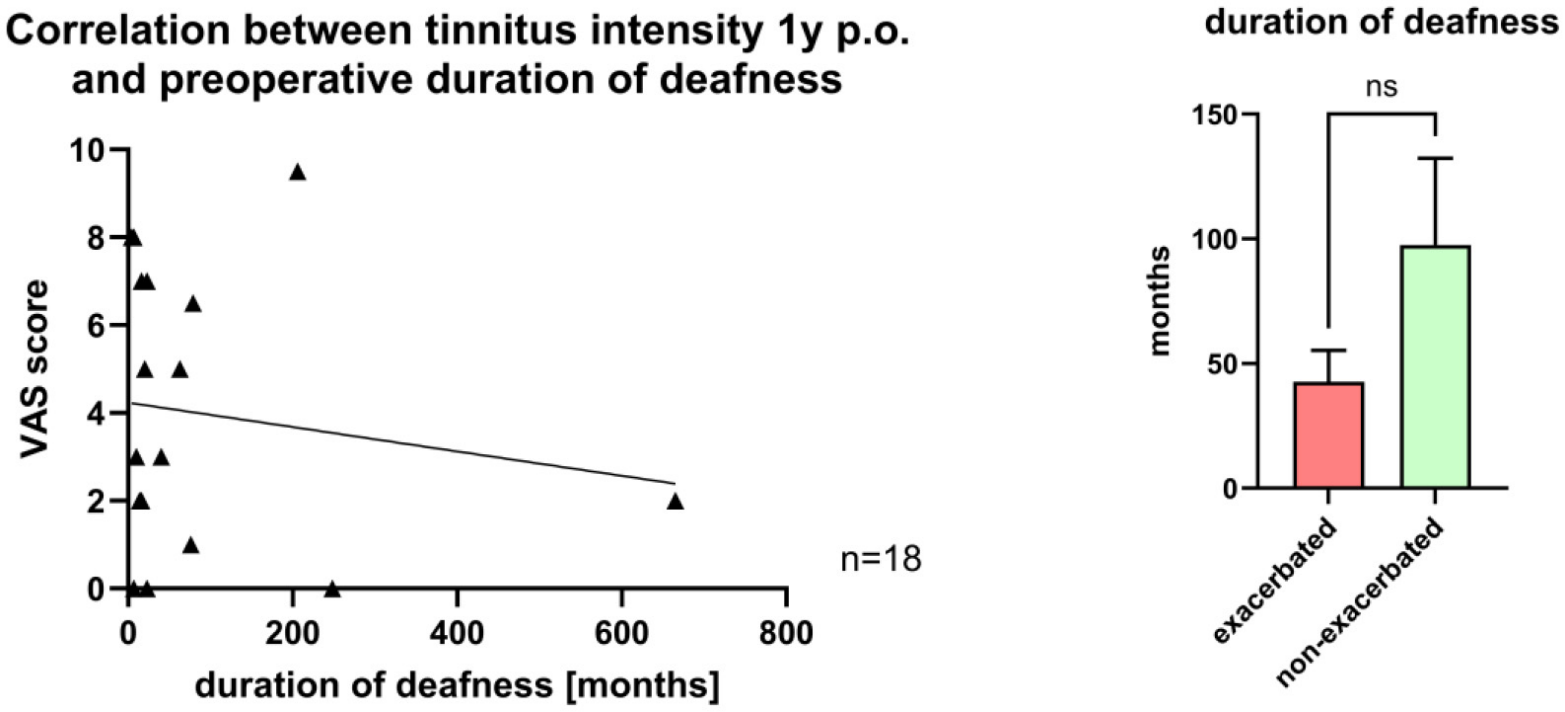
**Left**: Correlation between pre-operative deafness duration and tinnitus intensity one year post-operative. **Right**: Comparison of pre-operative deafness duration of patients with exacerbated tinnitus and non-exacerbated tinnitus 1 year post-surgery.

The mean pre-operative duration of deafness was 94 ± 173 months (range: 1–912 months). Patients with pre-operative residual hearing (in case of measuring PTA4 < 120 dB SPL AC; *n* = 18; 30%) were excluded in this analysis. No correlation was found between deafness duration and tinnitus intensity at 1 year post-surgery (Figure 5). Likewise, no significant difference in pre-operative deafness duration was observed between patients with and without tinnitus exacerbation at 1 year (exacerbation: 42.7 ± 12.7 vs. non-exacerbation: 97.50 ± 34.78 months; Figure 5).

#### Potential influence of etiology on tinnitus exacerbation

**Figure 6:**
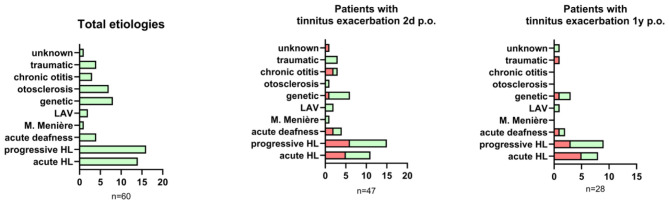
**Left**: Overview of total etiologies of hearing loss in this study. **Middle:** numbers of patients experiencing tinnitus exacerbation 2 days post-surgery (red) compared to patients without tinnitus exacerbation (green). **Right**: numbers of patients experiencing tinnitus exacerbation 1 year post-surgery (red) compared to patients without tinnitus exacerbation (green).

Etiologies of hearing loss are summarized in Figure 6, with progressive and acute hearing loss as the most common causes. Fisher's exact test compared patients with and without tinnitus exacerbation in regard to their etiology and revealed no significant association between etiology and tinnitus exacerbation at 2 days (*p* = 0.57) or 1 year post-surgery (*p* = 0.44; Figure 6).

#### Potential influence of pre-operative residual hearing on tinnitus exacerbation

**Figure 7:**
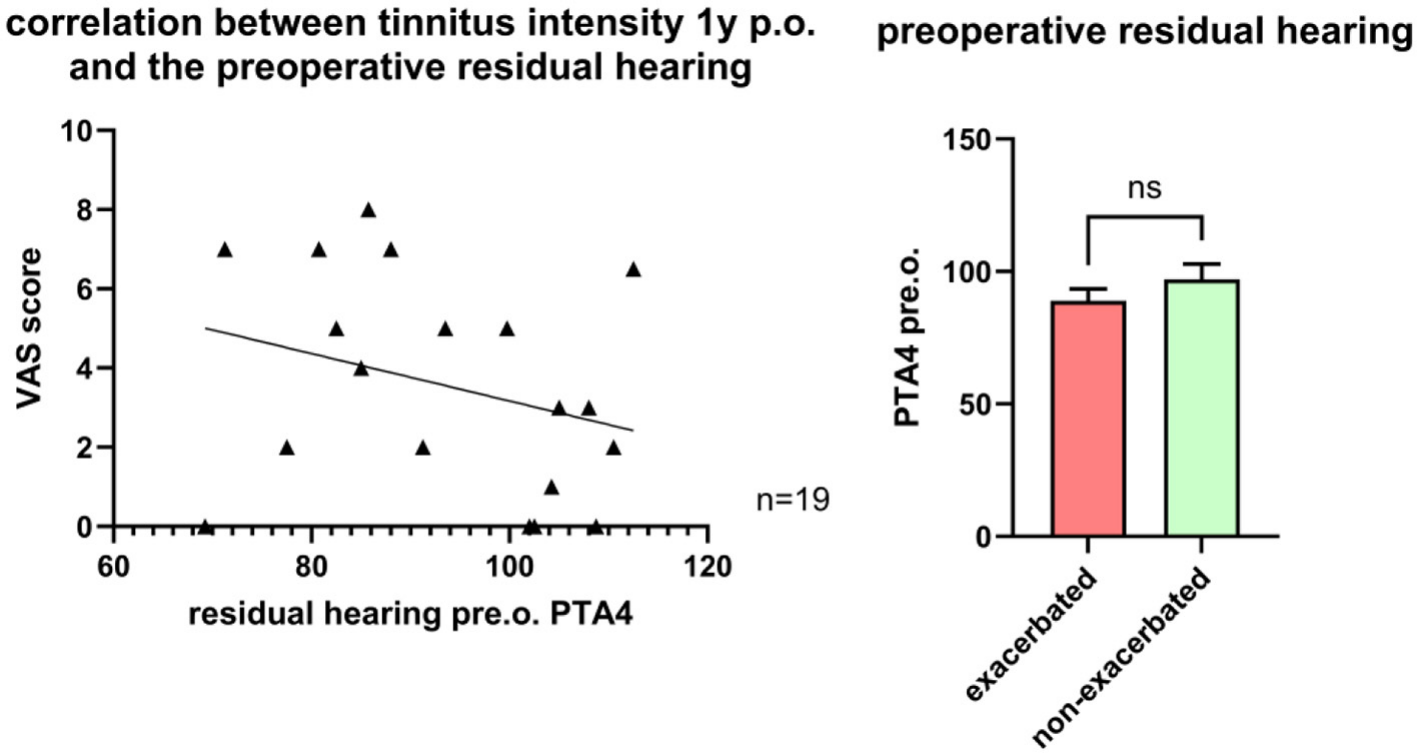
**Left**: Correlation between pre-operative residual hearing and tinnitus intensity one year post-operative. **Right**: Comparison of pre-operative residual hearing of patients with exacerbated tinnitus and non-exacerbated tinnitus 1 year post-surgery.

Pre-operative residual hearing was defined as a PTA4 <120 dB AC and was present in 43 patients (71%), with a mean PTA4 of 93.8 ± 12.8 dB SPL AC. No correlation was observed between tinnitus intensity and pre-operative or 1-year post-operative hearing levels (Figure 7). Similarly, pre-operative PTA4 did not differ significantly between patients with and without tinnitus exacerbation (89 ± 4.5 SPL AC dB vs. 97 ± 5.8 dB SPL AC; Figure 7). Among the 43 patients with residual hearing, 6 (14%) experienced partial loss within 4 weeks post-surgery, of whom 2 (33%) had tinnitus exacerbation.

### Morphological factors

Cochlear morphology was assessed using cochlear height and the product of distances A and B. No correlation was found between cochlear height and tinnitus intensity or burden at long-term follow-up. Patients with and without tinnitus exacerbation showed comparable cochlear height (4.123 ± 0.1 vs. 4.014 ± 0.1 mm) and distance A × B (69.24 ± 2.17 vs. 72.90 ± 1.95 mm^2^). Similarly, insertion depth (23.41 ± 1.1 vs. 21.97 ± 1.16 mm) and insertion angle (464.9 ± 28.69° vs. 416.7 ± 27.92°) did not differ significantly between patients with and without tinnitus exacerbation.

## Discussion

### Distributive factors

Although this study—consistent with previous findings in this cohort by Everad et al. (6)—confirms that the majority of CI recipients experience a significant reduction in tinnitus burden, a subset of patients still report persistent tinnitus, exacerbation, or even new-onset tinnitus, as also described by Olze et al. (2).

When comparing male and female patients with respect to tinnitus outcomes, no statistically significant differences were observed regarding the influence of CI implantation on tinnitus improvement, exacerbation, or intensity—neither in the short term (4 weeks post-operatively) nor in long-term follow-up at 1 and 2 years post-operatively. Although Mazurek et al. (25) emphasized the need for individualized psychotherapeutic and psychosomatic treatment approaches—accounting for sociocultural background, age, and gender, as also noted by Basso et al. (26) and Niemann et al. (27)—our study did not identify any significant differences in tinnitus outcomes after CI surgery based on age or gender.

Additionally, no significant correlation was found between the duration of deafness and post-operative tinnitus intensity or burden, suggesting that the length of auditory deprivation does not substantially influence tinnitus development following CI. Similarly, Czurda et al. (28) reported that patients with more than 20 years of hearing loss did not exhibit significantly different word recognition scores compared to those with shorter durations of deafness. However, other studies have demonstrated a significant impact of the duration of deafness on CI-related speech perception outcomes. For example, Bernhard et al. (29) performed a meta-analysis showing a negative correlation between the duration of deafness and post-operative speech perception. Liu et al. (30) found that shorter durations of deafness were associated with better tinnitus suppression and sound localization in a cohort of 26 single-sided deaf patients. Therefore, larger sample sizes and meta-analyses are still needed to examine the impact of deafness duration and degree of cochlear impairment on tinnitus intensity, burden, and development following CI surgery.

With regard to the different etiologies analyzed in this study, no significant influence on tinnitus burden or intensity was observed. While other studies (28, 31) have demonstrated a significant effect of etiology on post-operative speech perception—especially in cases of congenital or traumatic hearing loss, Menière's disease, or infectious etiologies such as chronic otitis media—the influence of etiology on tinnitus remains unclear. In our study, the number of patients with Menière's disease was too small to draw definitive conclusions. However, previous research (32, 33) has suggested that CI implantation may positively influence tinnitus burden in patients with Menière's disease. Nevertheless, these findings are based on studies with very small sample sizes.

The observed discordance between tinnitus intensity and tinnitus-related burden highlights the multifactorial nature of tinnitus perception. While tinnitus loudness may remain unchanged, reductions in tinnitus-related distress may reflect improved coping strategies, habituation, or central adaptation following auditory rehabilitation. This supports the concept that tinnitus intensity and tinnitus burden represent distinct but interrelated dimensions.

### Morphological and audiological factors

As previously reported in our study by Everad et al. (6), no significant influence of cochlear morphological parameters—such as distance A, distance B, or cochlear height—was found on post-operative tinnitus development. The current study extends these findings by showing that even in subgroup analyses of patients with tinnitus exacerbation, there were no significant anatomical differences or deviations in angular or linear insertion depth of the electrode array. The relationship between angular insertion depth and post-operative speech perception outcomes remains under debate. While some studies (34, 35) did not find a significant benefit of increased cochlear coverage on speech discrimination, others (36) reported improved outcomes, particularly with straight electrode arrays.

Another hypothesis tested in this study was whether traumatic insertions—potentially resulting in loss of pre-operative residual hearing—might induce or exacerbate tinnitus post-operatively. Although Everad et al. (6) found no increase in tinnitus intensity or burden in patients with confirmed traumatic insertion and scalar dislocation in post-operative radiological imaging, this is the first study to specifically examine the relationship between pre- and post-operative residual hearing and tinnitus outcomes. A loss of residual hearing was observed in six out of 47 patients (12%) at 4 weeks post-operatively. Among these, two patients (33%) experienced tinnitus exacerbation. This may indicate a trend or clustering effect, but no statistically significant correlation was found between post-operative residual hearing loss and tinnitus intensity. Moreover, there was no significant difference in post-operative residual hearing between patients with and without tinnitus exacerbation. Larger studies are needed to further explore these associations, ideally combining audiological outcomes, tinnitus burden, and psychosomatic assessments.

Strengths of this study include its prospective design with predefined follow-up intervals, the use of validated tinnitus assessment tools, standardized radiological assessment of cochlear morphology and electrode positioning, and the investigation of an underexplored but clinically relevant subgroup of patients experiencing tinnitus exacerbation after cochlear implantation.

### Limitations

This study is one of the few prospective investigations to explore the influence of distributive, morphological, and audiological factors on the development of tinnitus following CI surgery. However, the relatively small sample size is a key limitation. Particularly in subgroup analyses, the risk of type II error must be considered. Non-significant findings should therefore be interpreted with caution. Multivariable analyses were not performed due to the limited number of events, as this may have resulted in overfitting and unstable estimates. Follow-up attrition increased at later time points. This may introduce attrition bias, and long-term results should therefore be interpreted with caution. Pre-operative tinnitus and new-onset post-operative tinnitus may represent different underlying pathophysiological mechanisms. However, due to the limited number of patients with new-onset post-operative tinnitus, these entities were analyzed together, which should be considered a limitation of the study. Additionally, psychological and psychosomatic variables were not analyzed. Larger, multicenter studies are needed to validate these findings and to provide more comprehensive and generalizable insights. Also, it was not evaluated whether patients received tinnitus therapy before or after CI, which may have influenced the occurrence or perception of tinnitus.

## Conclusion

Two years after CI, 36% of patients who had reported tinnitus either pre- or post-operatively experienced an aggravation of tinnitus intensity, while 62% reported improvement and 17% showed no change in tinnitus-related distress. Importantly, no increased risk of tinnitus exacerbation at 1 year post-operatively was associated with gender, etiology of hearing loss, pre-operative residual hearing, post-operative residual hearing loss, duration of deafness, cochlear anatomy (including cochlear height and distances A and B), or electrode insertion depth and angle. These findings suggest that commonly considered distributive, anatomical, and audiological factors do not significantly predict long-term tinnitus exacerbation following CI.

## Data Availability

The raw data supporting the conclusions of this article will be made available by the authors, without undue reservation.

## References

[1] Arndt S Wesarg T Stelzig Y Jacob R Illg A Lesinski-Schiedat A . Influence of single-sided deafness on the auditory capacity of the better ear. HNO. (2020) 68(Suppl 1):17–24. doi: 10.1007/s00106-019-00739-6 31705300

[2] Olze H Ketterer MC Péus D Häußler SM Hildebrandt L Gräbel S . Effects of auditory rehabilitation with cochlear implant on tinnitus prevalence and distress, health-related quality of life, subjective hearing and psychological comorbidities: comparative analysis of patients with asymmetric hearing loss (AHL), double-sided (bilateral) deafness (DSD), and single-sided (unilateral) deafness (SSD). Front Neurol. (2023) 13:1089610. doi: 10.3389/fneur.2022.1089610 36712436 PMC9877424

[3] Péus D Pfluger A Häussler SM Knopke S Ketterer MC Szczepek AJ . Single-centre experience and practical considerations of the benefit of a second cochlear implant in bilaterally deaf adults. Eur Arch Otorhinolaryngol. (2021) 278:2289–96. doi: 10.1007/s00405-020-06315-x 32889623 PMC8165073

[4] Fries L Everad F Beck RL Aschendorff A Arndt S Ketterer MC . The influence of CI electrode array design on the preservation of residual hearing. Front Neurol. (2025) 16:1599369. doi: 10.3389/fneur.2025.1599369 40606125 PMC12213402

[5] Ketterer MC Shiraliyev K Arndt S Aschendorff A Beck R. Implantation and reimplantation: epidemiology, etiology and pathogenesis over the last 30 years. Eur Arch Otorhinolaryngol. (2024) 281:4095–102. doi: 10.1007/s00405-024-08568-2 38507077 PMC11266378

[6] Everad F Beck RL Aschendorff A Rauch AK Fries L Arndt S . Are tinnitus burden and tinnitus exacerbation after cochlear implantation influenced by insertion technique, array dislocation, and intracochlear trauma? Front Neurol. (2024) 15:1477259. doi: 10.3389/fneur.2024.1477259 39539659 PMC11557313

[7] Olze H Szczepek AJ Haupt H Zirke N Graebel S Mazurek B . The impact of cochlear implantation on tinnitus, stress and quality of life in postlingually deafened patients. Audiol Neurootol. (2012) 17:2–11. doi: 10.1159/000323847 21540584

[8] Knopke S Häussler S Gräbel S Wetterauer D Ketterer M Fluger A . Age dependent psychological factors influencing the outcome of Cochlear implantation in elderly patients. Otol Neurotol. (2019) 40:e441–53. doi: 10.1097/MAO.0000000000002179 30870379

[9] Häußler SM Knopke S Wiltner P Ketterer M Gräbel S Olze H . Long-term benefit of unilateral cochlear implantation on quality of life and speech perception in bilaterally deafened patients. Eur Acad Otol Neurotol. (2019) 40:e430–40. doi: 10.1097/MAO.0000000000002008 30870378

[10] Ketterer MC Aschendorff A Arndt S Hassepass F Wesarg T Laszig R . The influence of cochlear morphology on the final electrode array position. Eur Arch Otorhinolaringol. (2018) 275:385–94. doi: 10.1007/s00405-017-4842-y 29242990

[11] Ketterer MC Knopke S Häußler SM Hildenbrand T Becker C Gräbel S . Asymmetric hearing loss and the benefit of cochlear implantation regarding speech perception, tinnitus burden and psychological comorbidities: a prospective follow-up study. Eur Arch Otorhinolaringol. (2018) 275:2683–93. doi: 10.1007/s00405-018-5135-9 30229458

[12] Ketterer MC Häussler SM Hildenbrand T Speck I Peus D Rosner B . Binaural hearing rehabilitation improves speech perception, quality of life, tinnitus distress, and psychological comorbidities. Otol Neurotol. (2020) 41, e563–74. doi: 10.1097/MAO.0000000000002590 32068692

[13] Baguley DM Atlas MD. Cochlear implants and tinnitus. Prog Brain Res. (2007) 166:347–55. doi: 10.1016/S0079-6123(07)66033-6 17956799

[14] Blasco MA Redleaf MI. Cochlear implantation in unilateral sudden deafness improves tinnitus and speech comprehension: meta-analysis and systematic review. Am J Otol. (2014) 35:1426–32. doi: 10.1097/MAO.0000000000000431 24786540

[15] Quaranta N Wagstaff S Baguley DM. Tinnitus and cochlear implantation. Int J Audiol. (2004) 43:245–51. doi: 10.1080/14992020400050033 15357407

[16] Baguley D McFerran D Hall D. Tinnitus. Lancet. (2013) 382:1600–7. doi: 10.1016/S0140-6736(13)60142-7 23827090

[17] Liberman MC Kujawa SG. Cochlear synaptopathy in acquired sensorineural hearing loss: manifestations and mechanisms. Hear Res. (2017) 349:138–47. doi: 10.1016/j.heares.2017.01.003 28087419 PMC5438769

[18] Ramakers GGJ Kraaijenga VJC Smulders YE van Zon A Stegeman I Stokroos RJ . Tinnitus after simultaneous and sequential bilateral cochlear implantation. Front Surg. (2017) 4:65. doi: 10.3389/fsurg.2017.00065 29167796 PMC5682406

[19] Kloostra FJJ Arnold R Hofman R Burgerhof JGM van Dijk P. Models to predict positive and negative effects of cochlear implantation on tinnitus. Laryngoscope Invest Otolaryngol. (2019) 4:138–42. doi: 10.1002/lio2.224 30828631 PMC6383300

[20] Göbel G Hiller W. Tinnitus-Fragebogen:(TF); ein Instrument zur Erfassung von Belastung und Schweregrad bei Tinnitus; Handanweisung. Göttingen: Hogrefe, Verlag für Psychologie (1998)

[21] Escudé B James C Deguine O Cochard N Eter E Fraysse B. The size of the cochlea and predictions of insertion depth angles for cochlea implant electrodes. Audiol Neurootol. (2006) 11(Suppl 1):27–33. doi: 10.1159/000095611 17063008

[22] Ketterer MC Aschendorff A Arndt S Speck I Rauch AK Beck R . Radiological evaluation of a new straight electrode array compared to its precursors. Eur Arch Otorhinolaringol. (2021) 278:3707–14. doi: 10.1007/s00405-020-06434-5 33090276 PMC8382647

[23] Ketterer MC Rauch AK Beck RL Jakob TF Fries L Aschendorff A . The influence of electrode array design, scalar dislocation and insertion technique on postoperative vertigo in CI surgery - a prospective study. Eur Arch Otorhinolaryngol. (2025) 282:2367–72. doi: 10.1007/s00405-024-09147-1 39668219 PMC12055931

[24] Beck R Shiraliyev K Arndt S Rauch AK Aschendorff A Hassepass F . Scalar position, dislocation analysis and outcome in CI reimplantation due to device failure. Eur Arch Otorhinolaryngol. (2022) 279:4853–9. doi: 10.1007/s00405-022-07315-9 35226182 PMC9474456

[25] Mazurek B Böcking B Dobel C Rose M Brüggemann P. Tinnitus and Influencing Comorbidities. Laryngorhinootologie. (2023) 102:S50–8. doi: 10.1055/a-1950-6149 37130530 PMC10184670

[26] Basso L Boecking B Brueggemann P Pedersen NL Canlon B Cederroth CR . Gender-specific risk factors and comorbidities of bothersome tinnitus. Front Neurosci. (2020) 14:706. doi: 10.3389/fnins.2020.00706 33071718 PMC7539146

[27] Niemann U Boecking B Brueggemann P Mazurek B Spiliopoulou M. Gender-specific differences in patients with chronic tinnitus – baseline characteristics and treatment effects. Front Neurosci. (2020) 14:487. doi: 10.3389/fnins.2020.00487 32523506 PMC7261931

[28] Czurda R Wesarg T Aschendorff A Beck RL Hocke T Ketterer MC . Investigation of maximum monosyllabic word recognition as a predictor of speech understanding with cochlear implant. J Clin Med. (2024) 13:646. doi: 10.3390/jcm13030646 38337340 PMC10856473

[29] Bernhard N Gauger U Romo Ventura E Uecker FC Olze H Knopke S . Duration of deafness impacts auditory performance after cochlear implantation: a meta-analysis. Laryngoscope Investig Otolaryngol. (2021) 6:291–301. doi: 10.1002/lio2.528 33869761 PMC8035957

[30] Liu YW Cheng X Chen B Peng K Ishiyama A Fu QJ . Effect of tinnitus and duration of deafness on sound localization and speech recognition in noise in patients with single-sided deafness. Trends Hear. (2018) 22:2331216518813802. doi: 10.1177/2331216518813802 30509148 PMC6291880

[31] Janeschik S Teschendorf M Bagus H Arweiler-Harbeck D. Influence of etiologic factors on speech perception of cochlear-implanted children. Cochlear Implants Int. (2013) 14:190–9. doi: 10.1179/1754762812Y.0000000017 23510647

[32] Canzi P Manfrin M Perotti M Aprile F Quaglieri S Rebecchi E . Translabyrinthine vestibular neurectomy and simultaneous cochlear implant for Ménière's disease. Acta Neurochir. (2017) 159:123–30. doi: 10.1007/s00701-016-2996-9 27812817

[33] Mick P Amoodi H Arnoldner C Shipp D Friesen L Lin V . Cochlear implantation in patients with advanced Ménière's disease. Otol Neurotol. (2014) 35:1172–8. doi: 10.1097/MAO.0000000000000202 24366468

[34] Ketterer MC Aschendorff A Arndt S Beck R. Electrode array design determines scalar position, dislocation rate and angle and postoperative speech perception. Eur Arch Otorhinolaringol. (2022) 279:4257–67. doi: 10.1007/s00405-021-07160-2 34778920 PMC9363302

[35] James CJ Karoui C Laborde ML Lepage B Molinier CÉ Tartayre M . Early sentence recognition in adult cochlear implant users. Ear Hear. (2019) 40:905–17. doi: 10.1097/AUD.0000000000000670 30335668

[36] Breitsprecher TM Baumgartner WD Brown K Dazert S Doyle U Dhanasingh A . Effect of cochlear implant electrode insertion depth on speech perception outcomes: a systematic review. Otol Neurotol Open. (2023) 3:e045. doi: 10.1097/ONO.0000000000000045 38516541 PMC10950166

